# Segmentation-free Radon transform algorithm to detect orientation and size of tissue structures in multiphoton microscopy images

**DOI:** 10.1117/1.JBO.30.8.086001

**Published:** 2025-08-04

**Authors:** Danja Brandt, Anastasiia A. Nikishina, Anne Bias, Robert Günther, Anja E. Hauser, Georg N. Duda, Ingeborg E. Beckers, Raluca A. Niesner

**Affiliations:** a German Rheumatology Research Center, a Leibniz-Institute (DRFZ), Biophysical Analytics, Berlin, Germany; b Freie Universität Berlin, Dynamic and Functional in vivo Imaging, Department of Veterinary Medicine, Berlin, Germany; c Charité – Universitätsmedizin Berlin, Julius Wolff Institute for Biomechanics and Musculoskeletal Regeneration, Berlin, Germany; d Berlin University of Applied Sciences and Technology (BHT), Medical Physics, Departement of Mathematics – Physics – Chemistry, Berlin, Germany; e German Rheumatology Research Center, a Leibniz-Institute (DRFZ), Immune Dynamics, Berlin, Germany; f Charité - Universitätsmedizin Berlin, Department of Rheumatology and Clinical Immunology, Berlin, Germany

**Keywords:** Radon transform, fluorescence, second-harmonic generation, third-harmonic generation, collagen orientation, vessel orientation

## Abstract

**Significance:**

Understanding the structural organization of biological tissues is critical for studying their function and response to physiological and pathological conditions. *In vivo* imaging techniques, such as multiphoton microscopy, enable high-resolution visualization of tissue architecture. However, automated orientation analysis remains challenging due to imaging noise, complexity, and reliance on manual annotations, which are time-consuming and subjective.

**Aim:**

We present a Radon transform–based algorithm for robust, annotation-free structural orientation analysis across multimodal imaging datasets, aiming to improve objectivity and efficiency without introducing preprocessing artifacts.

**Approach:**

The algorithm employs a patch-based Radon transform approach to detect oriented structures in noisy images. By analyzing projection peaks in Radon space, it enhances small structures’ visibility while minimizing noise and artifact influence. The method was evaluated using synthetic and *in vivo* datasets, comparing its performance with human annotations.

**Results:**

The algorithm achieved strong agreement with human annotations, with detection accuracy exceeding 88% across different imaging modalities. Variability among trained raters emphasized the benefits of an objective, mathematically driven approach.

**Conclusions:**

The proposed method provides a robust and adaptable solution for structural orientation analysis in biological images. Its ability to quantify tissue component orientation without preprocessing artifacts makes it valuable for high-resolution, dynamic studies in tissue architecture and biomechanics.

## 1 Introduction

The structural organization of biological tissues provides critical insights into their functional properties and their responses to physiological and pathological conditions. The supportive tissue framework, with its specific orientation and anisotropies, is designed to fulfill diverse functions, adapt to varying conditions, and respond to external stimuli. A prime example is bone, where collagen fiber orientation plays a pivotal role in tissue behavior and is arranged in a mechanically meaningful manner. During bone injury, the reorganization of collagen and blood vessels influences healing and mechanical properties.^1^ The architecture of blood vessels is also essential as it ensures the supply of necessary nutrients for bone development and regeneration.^2^ In addition to its structural role in bone, collagen, the primary protein in the human body, also governs mechanical properties, such as tensile strength, and mediates cellular interactions.^3^^,^^4^ Disruptions in collagen alignment are hallmarks of pathological conditions, highlighting the importance of quantifying its orientation for biomedical insights.^5^^,^^6^

To better understand tissue organization and its continuous remodeling, dynamic *in vivo* studies are essential. These studies require advanced imaging techniques capable of capturing structural changes over time. With this goal in mind, intravital multiphoton imaging, which includes both fluorescence-based and harmonic generation-based methods, has significantly advanced the study of tissue architecture. Fluorescence imaging, for instance, provides high resolution and penetration depth through exogenous labeling, whereas harmonic generation techniques, such as second-harmonic generation (SHG) and third-harmonic generation (THG), enable label-free visualization of collagen fibers and other structural components.^7^^–^^9^ However, *in vivo* imaging data are often noisy and complex, posing significant challenges for the automated analysis of structural orientation and size. A key challenge is shot noise, which appears as abnormally bright pixel intensities relative to the signal. This noise can cause discontinuities in connected structures, making their identification more difficult.

To extract meaningful information from biological images, preprocessing techniques are used to reduce noise and enhance contrast. Image transformations such as the Fourier transform (FT) or wavelet transform (WT) are then applied to convert images into a domain that enables more effective analysis. FT, for example, decomposes images into frequency components, facilitating the assessment of structural anisotropy.^10^ This method has been applied to SHG images of collagen in the optic nerve head;^11^ tendons, sclera, cartilage;^12^^,^^13^ and the temporomandibular joint.^14^ However, FT is noise-sensitive and may obscure details in structures with multiple orientations. WT offers both spectral and temporal resolution.^15^ and the Curvelet transform (CT), a particular wavelet transform, extends this capability with directional sensitivity.^16^ One method that builds on the CT is the CT-FIRE algorithm, which integrates Curvelet-based decomposition with fiber extraction to enhance collagen analysis.^17^ Although effective, it remains sensitive to noise and requires extensive preprocessing, which can introduce artifacts.

Beyond transform-based methods, spatial and statistical techniques are also widely used.^18^ Spatial characterization includes segmentation, skeletonization, and region-based methods that leverage intensity distributions. Algorithms such as region growing^19^ and Otsu’s thresholding^20^ segment structures based on intensity and texture. Advanced techniques include random forest classifiers (e.g., Ilastik^21^) and convolutional neural networks (CNNs), which learn complex structures from annotated datasets. However, high-quality annotations are particularly challenging for collagen, where indistinct boundaries and subjective labeling introduce variability. Although CNNs have demonstrated strong performance for denoising—even with limited data—their application to orientation detection remains limited. To our knowledge, no generally applicable CNNs have been trained on *in vivo* datasets for this purpose likely due to the restricted availability of such annotated data.

For texture analysis, the gray-level co-occurrence matrix (GLCM) quantifies spatial relationships among pixel intensities, extracting features such as contrast and homogeneity.^22^^,^^23^ However, its reliance on intensity distributions makes it sensitive to noise, reducing its robustness in images with low signal-to-noise ratio (SNR).

These limitations become particularly critical in applications requiring high sensitivity to structural details and spatial integrity. Noisy datasets and indistinct structural borders complicate feature extraction and comparisons across imaging modalities, making it challenging to ensure consistency in capturing dynamic biological processes.

To address these challenges, we developed an annotation-free Radon transform–based algorithm designed to detect structures in noisy images while preserving spatial integrity. Originally introduced by mathematician Johann Radon in 1917,^24^ the Radon transform (RT) has been extensively studied and applied across various fields.^25^^–^^30^ Unlike Ref. 30, which applied the RT to the Fourier spectrum for collagen fiber orientation quantification, we applied it directly to the original image. By first splitting the image into patches, our approach enhances analysis and enables a more comprehensive characterization of all orientations within network-like structures. Requiring minimal preprocessing and manual tuning, the algorithm operates with a small set of parameters that remain largely independent of the input image type. We validated its versatility and robustness using multimodal imaging data, including 2PM and SHG images of blood vessels and collagen bundles, THG images of connective tissue, and synthetic datasets, demonstrating its broad applicability across diverse imaging modalities.

## 2 Methods

### 2.1 Two-Photon Microscopy Using LIMBostomy and Three-Photon Microscopy

The images used in this study were acquired using two different image acquisition methods. In the first method, two-photon microendoscopy was performed with the LIMBostomy implant described in Ref. 31 and a two-photon microscope similar to that detailed in Ref. 32. A GRIN endoscopic lens (length: ∼5.07  mm; diameter: 0.60 mm; model: NEM-060-10-10-850-S-1.0p, GRINTech GmbH, Jena, Germany) was mounted on a specialized holder surgically affixed to the femur of the mouse. This lens has a numerical aperture (NA) of 0.5 on the object side. Laser excitation (optical parametric oscillator 1100 nm wavelength, 80 MHz repetition rate) and focusing into the sample were achieved using a multi-immersion objective lens (10× magnification, NA 0.6, XLPLN10XSVMP, Olympus, Hamburg, Germany). Two-dimensional images were acquired with a resolution of 512×512  pixels, corresponding to a field of view of $500\times 500\;\mu m^{2}$. Qdots fluorescence in blood vessels was detected at 655±25  nm, and SHG of collagen fibers was detected at 525±25  nm. Detection was performed using photomultiplier tubes.

In the second method, three-photon microscopy (3PM) (optical parametric amplifier, 1650 nm wavelength, 2 MHz repetition rate) was used as described in Refs. 33 and 34, employing a water immersion objective lens (25× magnification, NA 1.05, XLPLN25XWMP2, Olympus, Hamburg, Germany). The THG signal was detected at a wavelength of 525±25  nm, using a photomultiplier tube. Imaging was performed at a resolution of 563×563  pixels, representing a field of view of $300\times 300\;\mu m^{2}$.

### 2.2 Algorithm Overview

This study introduces a Radon transform–based algorithm designed to analyze biological images, with a focus on detecting oriented structures and determining their size. We specifically examined collagen fibers (acquired via SHG), blood vessels (acquired via fluorescence in 2PM), and other oriented structures (acquired via 3PM). Given the distinct nature of these structures, it was essential to develop an approach capable of analyzing all of them without bias, ensuring robustness across various scenarios. To evaluate the algorithm’s performance, we tested it on synthetic datasets using binary images, where structure widths were precisely known, edges were sharply defined, and orientations were explicitly known, providing inherently unbiased comparison labels.

The output consists of a set of points, each defined by its x,y coordinates, orientation, and the width of the underlying structures at that location. The following sections detail the preprocessing steps, configurable parameters, core functionality of the algorithm, and the metrics used for performance evaluation.

### 2.3 Data Characteristics and Preprocessing

The algorithm was tested on 2PM, SHG, and THG images, with the acquisition modalities described in Sec. 2.1. Due to visible lens contours caused by the imaging technique,^31^^,^^35^ the 2PM and SHG images were cropped to the inscribed square within the lens, eliminating border effects associated with the circular lens shape. This resulted in image sizes of 300×300  pixels for 2PM and SHG data, whereas THG and synthetic images had sizes of 563×563  pixels and 350×350  pixels, respectively. To provide a more comprehensive evaluation and basis for comparison, the synthetic images were designed to mimic the structural features found in the real images. These include elongated structures with varying width-to-length ratios (represented by rectangles of different sizes), parallel bundles oriented in the same direction (represented by thin parallel lines), and wavy parallel structures also aligned in the same direction.

For computational efficiency, all images (2PM, SHG, and THG) were normalized to a 0 to 255 intensity range, converting the original 32-bit depth images. The synthetic images were binary, with pixel values ranging from 0 to 255.

To address Moiré pattern artifacts in the *in vivo* 2PM and SHG images and to reduce sharp edges in synthetic images, a Gaussian filter with a sigma value of 2 pixels was applied to all data.

Two key parameters need to be set manually: the patch size and the background threshold. The patch size depends on the size of the structures to be detected. The background threshold, relative to the normalized image, is based on the intensity range of the background or nonstructural elements.

### 2.4 Outputs

The algorithm outputs a list of points corresponding to locations on the detected structures. Each point is characterized by its x,y-coordinates, along with the orientation and width of the structure at that location. These points can be saved in a tabular format for statistical analysis, plotted on an image as markers, or visualized as oriented segments by assigning an arbitrary length to each point.

The coordinates of structures in an image can be used to identify nearby or corresponding structures in a paired image, allowing direct comparison of the orientations between the two images. This capability facilitates the study of structural reorganizations in different tissues imaged with different modalities, as demonstrated in the LIMBostomy data.

The implementation is carried out in Python 3.9, with the Radon transform computation relying on the radon function from the scikit-image library.

### 2.5 Metrics

The performance of the proposed algorithm was evaluated by comparing the detected positions, orientations, and widths of the structures with the corresponding values from both synthetic and real image datasets.

Synthetic data were generated using Adobe Illustrator v27.9. For the rectangles and lines, the exact orientation, width, and label (structure number) were recorded in a table, serving as the ground truth for comparison. For the wavy structures, where the angle varies slightly at each point along the sinusoidal wave, the orientations at the coordinates of the detected points were labeled and recorded in the table. The evaluation process involved two steps:

Location detection accuracy: The first step was to verify whether the detected points were within the boundaries of the corresponding structures. If the pixel value at the detected point’s coordinates was 255, it was considered a correct detection; otherwise, it was marked as incorrect.

Comparison of detected attributes: On the base of the location detection, the algorithm’s output for orientation and width was compared with the ground truth values in the label table. The comparison criteria were as follows:

• The orientation was considered correct if the detected angle was within a 5% tolerance, which corresponds to 9 deg for a 180 deg angle range, of the ground truth. This threshold balances precision and robustness, allowing minor variations while ensuring the detected orientations closely match the synthetic ground truth.
• The width was considered correct if the detected value was within a 2-pixel difference from the ground truth. Given the discrete nature of images, 2 pixels is a practical minimum tolerance that maintains both accuracy and robustness.

The performance of the real data was evaluated by comparing the algorithm’s outputs with the labels provided by five trained raters. The labeling process involved the following steps:

1. The raters manually drew a mask on the image, marking the structures of interest.
2. The raters were then provided with the image containing the detected points. For each point, they marked the corresponding orientation and width of the underlying structure.

For evaluation, the following steps were taken:

Location detection accuracy: The first step was to verify whether the detected points were within the boundaries of the manually drawn mask. If a point fell within the mask, it was marked as correct. As the algorithm appeared more accurate in some cases than the hand-labeled masks, points outside the mask were re-evaluated by the raters. Each rater reviewed the points marked as incorrect, determining whether they were truly incorrect or should be included among the correct ones. An example of this evaluation process is provided in Fig. S1 in the Supplementary Material.

Comparison of detected attributes: Similar to synthetic data, the detected orientation and width were compared with the ground truth values provided by the raters. Tolerance values for angles and widths were calculated using the standard deviation of the raters’ labels. For angles, the circular nature of the data was taken into account using circular statistics to ensure accurate tolerance estimation.

## 3 Algorithm

### 3.1 Radon Transform

The Radon transform (RT), widely recognized as the mathematical foundation of tomographic reconstruction, is also highly effective for detecting the orientation of lines and features within an image. When applied to an image, the RT projects the image’s pixel values along multiple angles, generating a series of projections. Peaks within these projections correspond to prominent features, such as lines, in the image. Analyzing the position of these peaks reveals the direction of the detected lines.

**Fig. 1:**
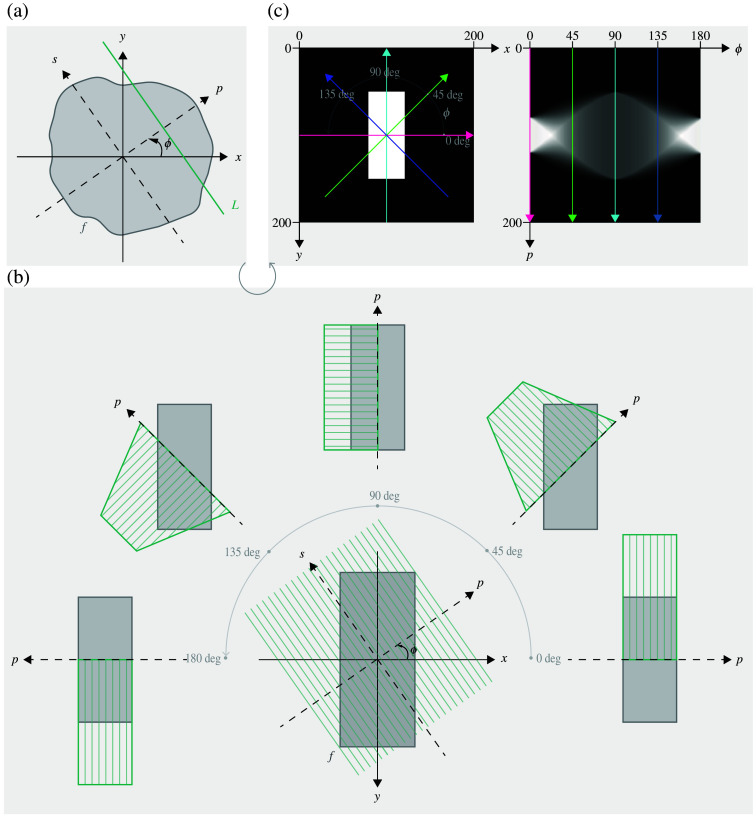
Radon transform (RT) principle. (a) The RT maps a function f(x,y) from the spatial domain (x,y) to the Radon space (ϕ,p). For any line L in the plane, the RT of f is defined as the projection or line integral of f along all possible lines L. A line L can be expressed in polar form as p=x cos(ϕ)+y sin(ϕ), where p is the perpendicular distance from the origin, and ϕ is the angle of its normal with the positive x-axis. In a rotated coordinate system (p,s), where s is aligned with L, the transformations are x=p cos(ϕ)−s sin(ϕ), y=p sin(ϕ)+s cos(ϕ). The line integral along an increment ds along L is thus a function of p and ϕ. (b) Projection of a rectangle at selected angles (0, 45, 90, 135, and 180 deg). For each angle ϕ, pixel intensities are summed along the direction perpendicular to p (thin green lines), forming projection curves (thick green lines) that vary with ϕ. (c) The sinogram is generated by projecting a function across all angles in [0,π]. On the right, the sinogram of the left image is shown. The p-axis maximum equals the shortest side of the input image, whereas the ϕ-axis on the right represents the rotation angle. Projection lines at 0, 45, 90, and 135 deg are displayed on both panels. The pixel intensities along each p-line in the sinogram represent summed pixel intensities along the direction perpendicular to p in the original image.

A clear, two-dimensional definition of the RT was presented by Deans in 1983.^25^ Referring to Fig. 1(a), let (x,y) denote Cartesian coordinates in the plane, and consider an arbitrary function f defined on a domain $D\subset R^{2}$. For any line L in the plane, the Radon transform of f is the mapping defined by the projection or line integral $\overset{˘}{f}$ of f along all possible lines L, assuming the integral exists. Specifically, it is defined as

$$\overset{˘}{f}=RT(f)=\int_{L}f(x,y)ds,$$ (1)

where ds is an element of length along L. If the line L is described in polar form as

p=x cos(ϕ)+y sin(ϕ), (2)

then the integral (1) depends on the parameters of p and ϕ, leading to the expression

$$\overset{˘}{f}(p,\phi )=\int_{L}f(x,y)ds=\int_{L}f(p\;\cos\;\phi -s\;\sin\;\phi ,p\;\sin\;\phi +s\;\cos\;\phi )ds,$$ (3)

where the coordinates (x,y) can be expressed in terms of a rotated coordinate system (p,s) as x=p cos(ϕ)−s sin(ϕ) and y=p sin(ϕ)+s cos(ϕ). For functions f that vanish outside a bounded domain D, the integral may have finite limits.

Figure 1(b) illustrates an example of a rectangle and its projections along selected angles (0, 45, 90, 135, and 180 deg). For each angle ϕ, the projection on p-axis is calculated by summing the pixel intensities along the direction of L.^36^ When all possible lines L(p,ϕ) are considered for p∈R and ϕ∈[0,π], the result is a comprehensive mapping known as a sinogram, as shown in Fig. 1(c). This sinogram encapsulates the projections of the rectangle across all rotation angles, providing a complete representation of its structural information in Radon space.

### 3.2 Algorithm Design for Orientation and Size Analysis in Noisy Images

**Fig. 2:**
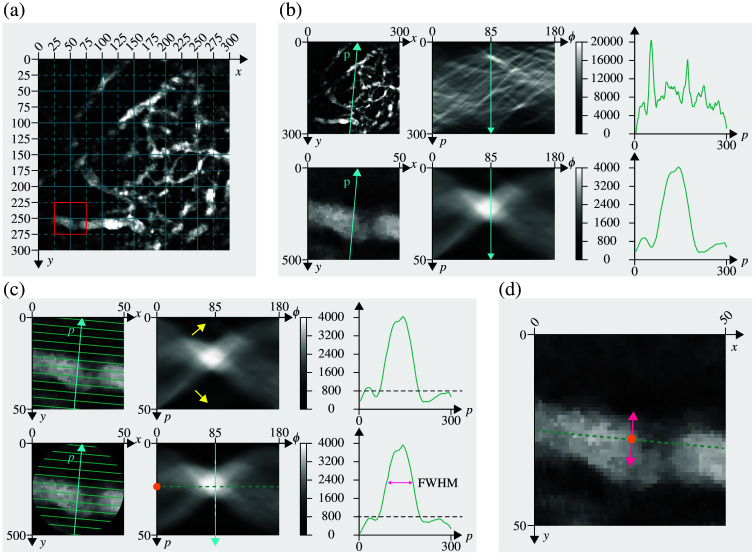
Algorithm design. (a) Illustration of the patch-based grid structure. Solid lines indicate the initial patch divisions, whereas dashed lines represent the overlapping patches created by shifting each patch horizontally and vertically by half its width and height. The patch within the red square is used as an example. (b) Radon transform applied to both the entire image and an individual patch. On the left, the original image (300×300  pixels, 1  pixel=0.976  μm) is shown in the first row, with a 50×50  pixel patch in the second row. The projection axis p, oriented at 85 deg, is displayed in cyan. In the center, the Radon transform (or sinogram) of the entire image and the patch is shown, with the same projection axis p highlighted in cyan in the (p,ϕ) coordinate system. On the right, the projection curves onto the p-axis at 85 deg. The ordinate of the projection plot represents the pixel intensities of the sinogram along p-axis. The projection plot of the entire image reveals multiple noisy peaks from various structures, complicating automated analysis. By contrast, the projection curve of the patch shows a distinct, nearly noise-free peak, facilitating easier analysis. (c) Steps of the algorithm. On the left, a patch is shown before (top row) and after (bottom row) the application of a circular window. The effects of image shape on the sinogram are highlighted by yellow arrows in the first row (center) and in the corresponding projection plot (right). Using a circular window shifts the extremes of the projection plot toward an intensity value of 0, eliminating peaks above 800 caused by image corners, as shown in the bottom row. After applying the window to the input image and computing the sinogram, the sinogram is analyzed to determine the coordinates of the predominant peak, the p- and ϕ-coordinates. The full width at half maximum (FWHM) of the detected peak, indicated by the magenta arrow in the plot, defines the width of the structure generating it. (d) Estimating the structure’s position in the input image: The position is approximated by converting the detected polar coordinates back to the Cartesian plane (x,y), assuming s=0, as indicated by the yellow dot in the image. The structure’s orientation in the input image, obtained by shifting the detected peak’s ϕ-coordinate by 90 deg, is represented by the green dashed line. The FWHM is shown in magenta.

Drawing from this foundation, the proposed algorithm aims to identify oriented structures in the input image by analyzing the peaks in the Radon space. The core of this algorithm lies in the step preceding the computation of the RT. Instead of computing the RT over the entire image, the image was divided into smaller patches. To ensure full coverage, each patch was then shifted horizontally and vertically by half its width and height, creating an overlapping set of patches that captured previously uncovered regions. Figure 2(a) provides a visual representation of this grid, where solid lines indicate the initial patch divisions, and dashed lines represent the overlapping patches.

This approach has several key advantages. First, when applying the RT to the entire image, only large structures tend to produce prominent peaks, whereas smaller structures are often classified as background. By dividing the image into patches, we enhanced the visibility of smaller structures, making them easier to detect. In addition, as the RT generates peaks by summing pixel intensities along a specific direction, overexposed regions—frequent in fluorescent and harmonics generation images and typically round rather than elongated—can distort peak formation. The patch-based approach mitigates this issue by reducing the influence of overexposed areas on the overall results. Furthermore, this patch-based strategy yielded a large number of validation samples (at least 196 patches per image), which helped strengthen the robustness of our performance evaluation despite the limited number of manually labeled images. Figure 2(b) illustrates examples of the Radon transform applied to both the entire image and an individual patch, along with the corresponding projection curves onto the p-axis oriented at 85 deg. The projection plot of the entire image exhibits multiple noisy peaks caused by various structures, making automated analysis challenging. By contrast, the projection curve of the patch displays a distinct, nearly noise-free peak, significantly simplifying the analysis.

Setting the background threshold value is justified by the need to focus on patches containing relevant structures and to streamline the analysis. Patches where >80% of the pixels have intensity values below this threshold are excluded from the analysis. After this initial filtering, the Radon transform is applied to the remaining patches. To minimize artifacts caused by the boundaries of a rectangular image, a circular mask is applied, assuming the image extends in a circular shape. This approach prevents edge distortions during rotation and ensures smoother projections. As shown in Fig. 2(c), the effects caused by the image shape are highlighted by yellow arrows in the first row. This effect is also evident in the projection plots of the sinograms: the curve exhibits values above zero at the extremes, and two intensity peaks above 800 can be observed if the image is not windowed.

Each sinogram is then analyzed to identify the dominant peak, and its position in the sinogram image is returned as coordinates. Specifically, the peak’s location along the p-axis [represented by the yellow dot on the bottom line of Fig. 2(c)] and its position along the ϕ-axis are determined. With a 90 deg shift, the latter corresponds to the orientation of the structure in the input image, represented by the green dashed line in Fig. 2(d). The returned coordinates can then be used to determine the position of the structure in the input image, assuming the ordinate is s=0. The location of the yellow point in the input image is shown in Fig. 2(d). In addition, the full width at half maximum (FWHM) of the detected peak, as shown in the plot in Fig. 2(c), is used to define the width of the structure generating it.

To assess the accuracy of the predicted peak, the intensity profile along the chord of the circular mask, which is oriented according to the detected angle and passes through the detected point, is calculated [green dashed line in Fig. 2(d)]. If all points on this line exceed the threshold value, the structure is considered continuous and uninterrupted. The center points, orientations, and widths that pass this final filtering step are returned as the output of the algorithm. A flowchart summarizing the complete algorithmic workflow is provided in Fig. S2 in the Supplementary Material. The computation of 196 patches, each of size 40×40  pixels, using an Intel Core i7 processor takes 2.4 s, making it an efficient and fast method. In comparison, manually annotating a single image takes ∼1  h.

## 4 Results

### 4.1 Benchmarking Algorithm Performance on Synthetic Data

#### 4.1.1 Synthetic data

**Fig. 3:**
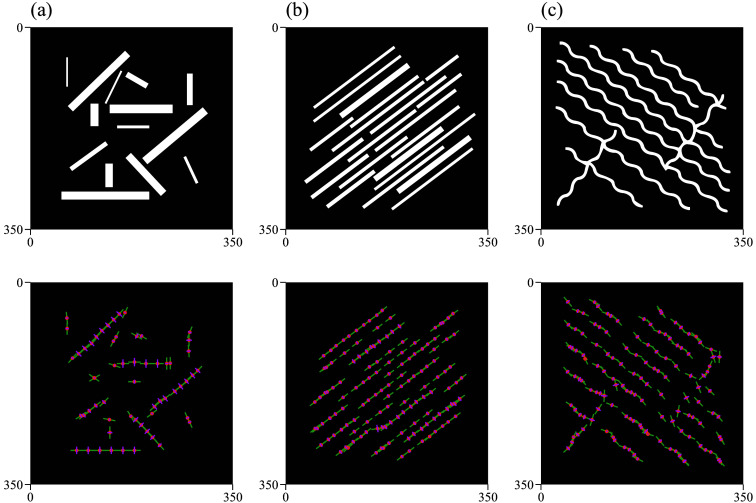
Synthetic data examples and corresponding algorithm output. (a) Shapes with varying widths and orientations. (b) Shapes with similar widths aligned at the same angles. (c) Wavy structures predominantly oriented in the same direction. Below is an overview of the algorithm’s results on each dataset.

We generated three synthetic data examples to replicate structural patterns observed in real images. The first dataset contains shapes with varying widths and orientations, the second consists of shapes with similar widths aligned at the same angles, and the third features wavy structures predominantly oriented in the same direction. In the first case, the widths range from 3 to 15 pixels, which are representative of the vessel widths observed in our real data. An overview of the synthetic data and the corresponding algorithm output is presented in Fig. 3.

#### 4.1.2 Influence of patch size on detection accuracy

**Fig. 4:**
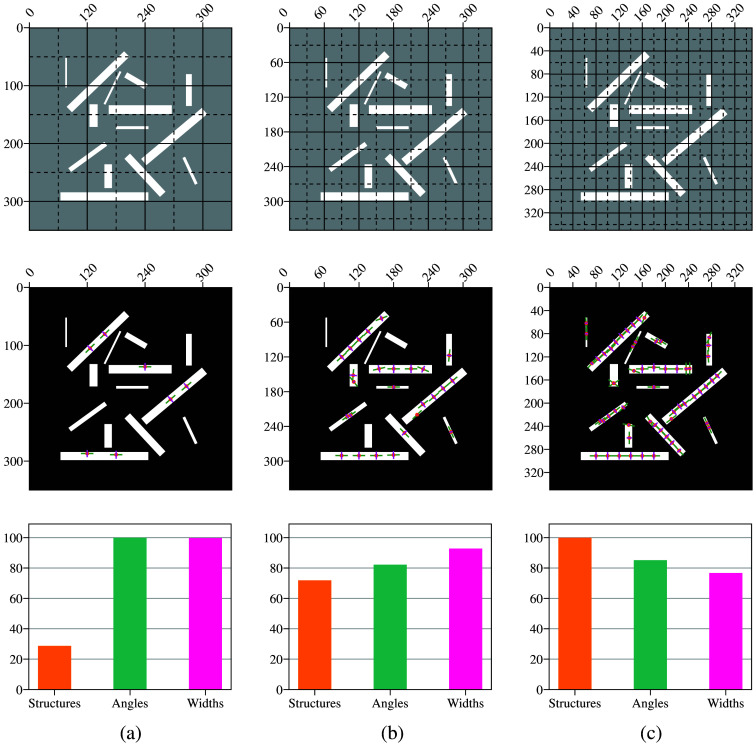
Effect of patch size. From top to bottom, patch division is shown with continuous lines representing original patches and dashed lines indicating overlapping patches. The algorithm’s output is visualized by segments, with green indicating points, cyan for orientation, and magenta for width. A histogram displays the percentage of correct points, angles, and widths. (a) For 36 patches of size 100×100  pixels, the detection rate reached 28.5%, whereas orientation and width accuracies both reached 100%. (b) For 100 patches of size 60×60  pixels, the detection rate improved to 71.4%, with orientation and width accuracies at 81% and 92%, respectively. (c) For 256 patches of size 40×40  pixels, the detection rate reached 100%, whereas orientation and width accuracies were 85% and 76.6%, respectively. Smaller window sizes, although effective at capturing individual structures, may introduce inaccuracies along the edges of large, sharp binary structures, particularly in orientation and width estimation.

To illustrate how patch size affects the output, we applied the algorithm to an artificially generated 350×350  pixel image using patch sizes of 100, 60, and 40 pixels. The selected image was the most complex among the generated options, featuring structures with varying widths and orientations to ensure a robust evaluation. Patch positions are illustrated in the first row of Fig. 4, where continuous lines represent the initial patch division and dashed lines indicate overlapping patches. A total of 36, 100, and 256 patches are generated for patch sizes of 100, 60, and 40 pixels, respectively. The 100-pixel patch size was selected to ensure a sufficient number of patches for analyzing global structures while preserving structural detail. By contrast, the smallest size was selected based on the maximum observed structure width of 15 pixels. A 40-pixel patch size was chosen as it spans approximately twice this width (15×2) while compensating for portions lost after applying the circular mask. A 60-pixel patch size served as an intermediate option to balance fine detail capture with accuracy in larger structures.

As shown in the second row of Fig. 4, larger patches effectively detect long and wide structures, achieving 100% accuracy in orientation and width. However, they fail to identify smaller structures, leading to a total detection rate of just 28.5%. Reducing the patch size enables the identification of even the smallest structures, improving the detection rate to 71.4% for a 60-pixel patch and achieving a perfect detection rate of 100% with a 40-pixel patch.

Conversely, smaller patch sizes can introduce inaccuracies along the edges of large, sharp binary structures, particularly in orientation and width estimation. These limitations result in angle and width accuracies of 81% and 92%, and 85% and 76.6%, respectively, as shown in the last row of Fig. 4.

#### 4.1.3 Algorithm accuracy for various geometries

**Fig. 5:**
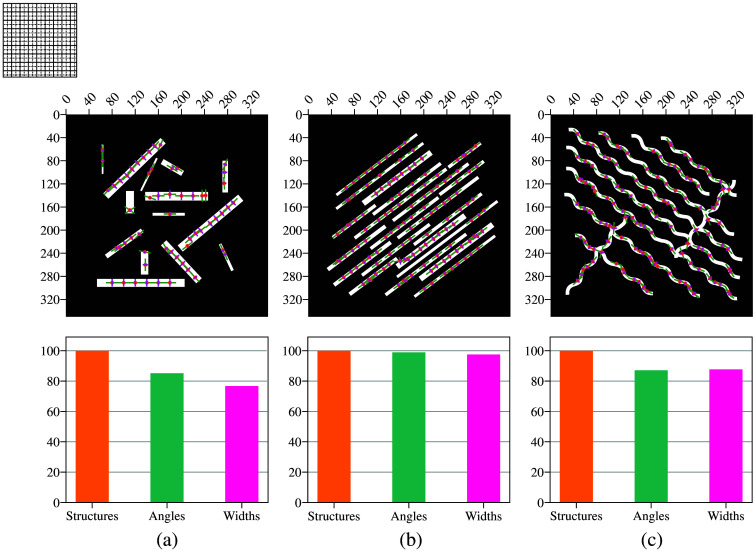
Results overview on synthetic data. A patch size of 40×40  pixels was used for images (a), (b), and (c). (a) Elongated structures with varying width-to-length ratios: the detection rate was 100%, whereas orientation and width accuracies were 85% and 76.6%, respectively. (b) Parallel bundles oriented in the same direction: the detection rate was 28.5%, with orientation and width accuracies of 99.2% and 97%, respectively. (c) Wavy parallel structures aligned in the same direction: the detection rate was 28.5%, whereas orientation and width accuracies were 86.9% and 87.7%, respectively. Simpler structures with consistent orientation achieved better results.

In addition to the image analyzed in Sec. 4.1.2, the algorithm was tested on two additional synthetic images, using a consistent patch size of 40 pixels for both tests. The images and corresponding results are shown in Fig. 5. For Fig. 5(b), where the structures are simpler and the orientation is consistent along their entire length, the algorithm achieved an accuracy of 99.2% for angles and 97% for widths. By contrast, in Fig. 5(c), angle detection proved more challenging due to the varying orientation at each point along the sinusoidal curve. Nevertheless, the accuracy for the detected angles and widths was 86.9% and 87.6%, respectively. Remarkably, the structure detection accuracy reached 100% across all images.

### 4.2 Evaluating Structure Orientation and Size in Original *In Vivo* Data

#### 4.2.1 *In vivo* multiphoton images of tissue structures

**Fig. 6:**
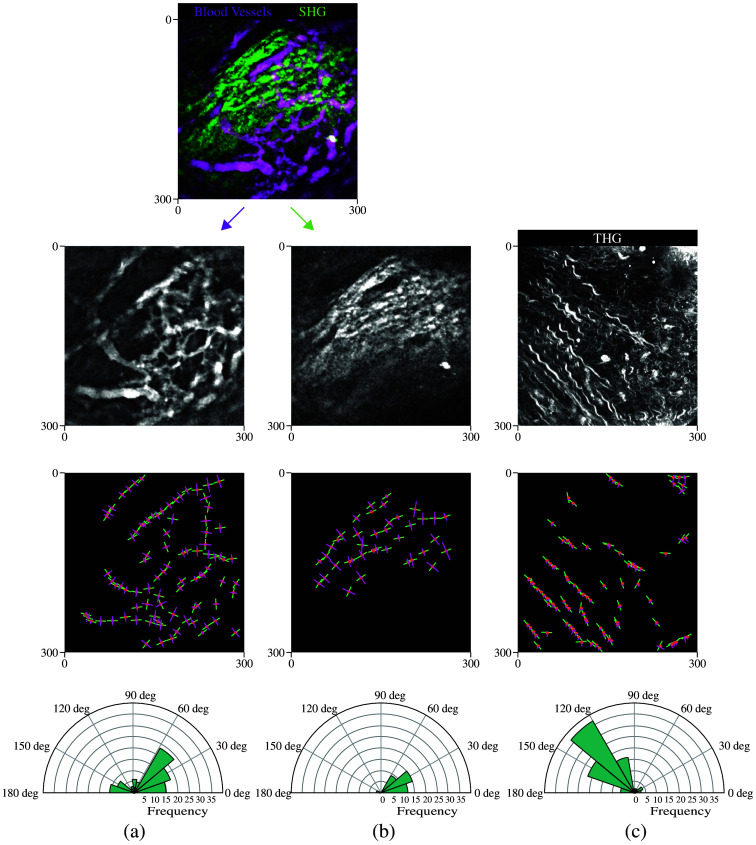
*In vivo* multiphoton imaging data of tissue architecture and corresponding algorithm output. (a) Fluorescence images of blood vessels using two-photon microscopy (2PM). (b) SHG images of collagen fibers. (c) THG images of connective tissue. The first row presents the merged and co-registered 2PM and SHG images. Below, an overview of the algorithm’s results for (a), (b), and (c) is shown, with the last row displaying polar histograms of the detected orientations for each image. For 2PM and SHG, 1  pixel=0.976  μm; for THG, 1  pixel=0.533  μm.

We acquired coregistered fluorescence images of blood vessels using 2PM and SHG images of collagen fibers using the LIMBostomy technique,^31^ along with the THG images of connective tissue. The LIMBostomy data were cropped to a square shape before analysis to avoid discontinuities at the lens border. The algorithm was applied to the data using a patch size of 40 pixels to extract the orientation and width of the structures. An overview of the images and the results is shown in Fig. 6.

#### 4.2.2 Evaluation of signal-to-noise ratio before and after Gaussian filtering

To assess the impact of Gaussian filtering on image quality, we evaluated the SNR before and after applying a Gaussian filter with a sigma value of 2 pixels. The SNR was computed using the following equation:

$$\mathrm{SNR}=\frac{\mu_{\text{signal}}-\mu_{\text{background}}}{\sigma_{\text{signal}}},$$ (4)

where $\mu_{\text{signal}}$ and $\mu_{\text{background}}$ represent the mean values of the signal and background, respectively, and $\sigma_{\text{signal}}$ is the standard deviation of the signal.

Table 1 presents the SNR values measured before and after filtering for 2PM, SHG, and THG. For each image, five regions of interest (ROIs) were selected for the signal and five for the background. The mean values from these ROIs were used to compute the SNR. The calculated SNR values are expressed in decibels (dB) using the following logarithmic transformation

$${\mathrm{SNR}}_{\mathrm{dB}}=10\cdot \log_{10}(\mathrm{SNR}).$$ (5)

**Table 1:** SNR before and after Gaussian filtering for different imaging modalities.

Data	SNR (original) (dB)	SNR (filtered) (dB)
2PM	6.95	7.89
SHG	4.88	6.91
THG	5.44	8.1

#### 4.2.3 Analysis performance of trained raters

To assess the algorithm’s performance, five trained raters manually annotated the images. The resulting labels exhibited variation reflecting the complexity of the images. Due to this variability, it was first necessary to quantify the degree of disagreement among raters. To achieve this, the standard deviation of both the angle and width labels was calculated for each point across raters. Then, for each image, the mean standard deviation was computed separately for angle ($\sigma_{\text{angles}}$) and width ($\sigma_{\text{widths}}$) labels. These mean standard deviations were used to define tolerance values, representing the acceptable range within which an angle or width value was considered correct. The calculated tolerance values for each image are presented in Table 2.

**Table 2:** Tolerance values.

Data	$\sigma_{\text{angles}}$	$\sigma_{\text{widths}}$
2PM	27.43	8.82
SHG	29.96	18.12
THG	29.12	13.55

Using these tolerance values, the variability among labels was quantified by selecting one rater as the reference. To evaluate the masks, each mask was compared with the reference rater’s mask using the intersection over union (IoU). For each point, the correctness of the angle and width labels was determined, as described in Sec. 2.5: the reference rater was treated as the ground truth, and an angle or width was considered correct if it fell within the reference value ± its standard deviation.

**Fig. 7:**
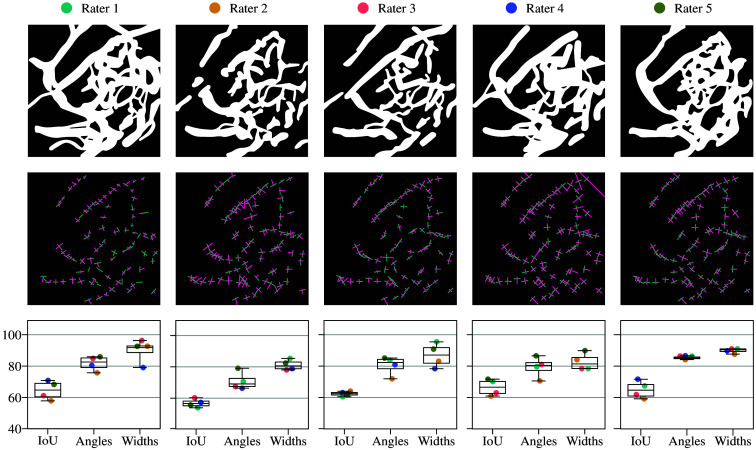
Variability in rater annotations for the 2PM data. The first row displays the masks drawn by different raters, whereas the second row shows the orientation (green lines) and width (magenta) assigned by each rater. Differences in annotations are evident, even for structures that are relatively easier to detect, such as blood vessels, due to the complexity of the image. In each column, the corresponding rater is treated as the ground truth. The last row presents the evaluation results of the other raters relative to the reference rater, using the intersection over union (IoU) metric for the masks and the differences between the orientations and widths assigned by the reference rater and the other raters.

This process was repeated for each rater, with each one taking turns as the reference while being compared against the others. As an example of this evaluation, the 2PM data are presented in Fig. 7. The first row illustrates the visible differences between the masks drawn by each rater, with each rater assigned a different color. In each column, the corresponding rater was treated as the ground truth, whereas the results of the other raters are shown in the plot in the last row. The evaluations for the SHG and THG data can be found in the Figs. S3 and S4 in the Supplementary Material.

**Fig. 8:**
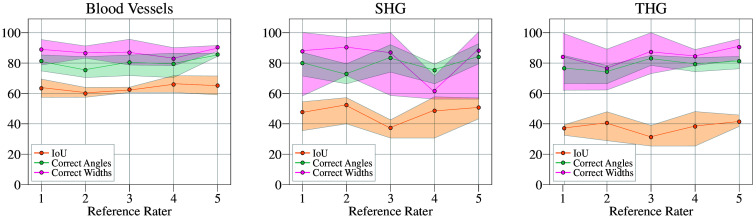
Mean percentage of correct classifications across raters, with each rater sequentially serving as the reference. The x-axis represents the selected reference rater, whereas the y-axis shows the mean percentage of correct classifications among the remaining raters, with the corresponding minimum and maximum limits.

The overall results of this analysis are presented in Fig. 8, where the x-axis represents the selected reference rater, whereas the y-axis displays the mean percentage of correct classifications across the remaining raters, along with the corresponding minimum and maximum limits.

Following the initial evaluation of variability among raters, a second evaluation was conducted to refine the accuracy of the detected points. After this re-assessment, only a small number of additional points were included as correct detections. This refinement had a minimal impact on the overall accuracy but ensured that the most precise assessment was achieved. The final adjusted point detection accuracy is summarized in Table S1 in the Supplementary Material, which presents the number of initially detected correct points alongside the additional points verified as correct after re-evaluation.

#### 4.2.4 Algorithm performance compared with trained raters

**Fig. 9:**
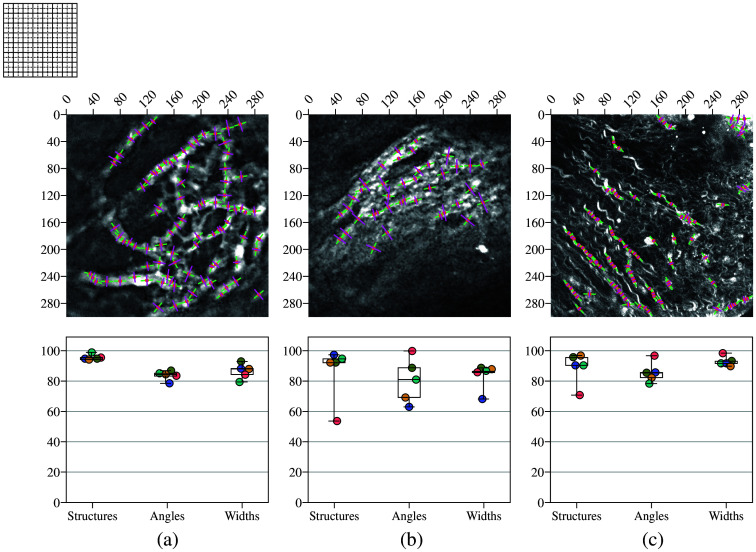
Comparison of algorithm-detected structures with rater annotations across different imaging modalities, with each rater represented by a point in a different color. (a) 2PM images of blood vessels, where the algorithm achieved a mean structure detection accuracy of 96.2%, with angle and width detection accuracies of 83.9% and 86.2%, respectively. Strong agreement was observed among raters for segmentation masks and angle measurements, whereas width estimations showed greater variability due to the lack of sharp vessel boundaries. (b) SHG images of collagen bundles, where the algorithm reached a mean structure detection accuracy of 89.7%, with 79.1% and 84.8% accuracy for angle and width detection, respectively. Higher discrepancies among raters were observed, particularly for orientation labels, due to the subjective nature of collagen bundle delineation. (c) THG images of connective tissue, where the algorithm achieved a mean structure detection accuracy of 88.8%, with 85.7% accuracy for angle detection and 92% for width detection. Width measurements showed the highest consistency among raters, attributed to the well-defined structure boundaries, whereas angle measurements exhibited some discrepancies due to the sinusoidal nature of the tissue. For 2PM and SHG, 1  pixel=0.976  μm; for THG, 1  pixel=0.533  μm.

The algorithm was tested on real images, and the outputs were compared with the labels assigned by each rater. A consistent patch size of 40 pixels was used for all images. The images and corresponding results are presented in Fig. 9. For the 2PM data representing blood vessels [Fig. 9(a)], the mean accuracy of the detected structures reached 96.2%, whereas the accuracy for angle and width detection was 83.9% and 86.3%, respectively. As illustrated in the boxplot, raters showed strong agreement on segmentation masks and angle labels likely due to the relatively simple nature of these images, where structures can easily be segmented and their orientations clearly identified. By contrast, width measurements exhibited greater discrepancies likely due to the lack of sharp vessel boundaries. For the SHG data representing collagen bundles, the mean accuracy of the detected structures reached 89.7%, with angle and width detection accuracies of 79.1% and 84.8%, respectively. The complexity of these images makes it difficult to define precise masks as the distinction between bundles and background is often subjective, leading to variability among raters. Interestingly, orientation labels showed high discrepancies. Although a dominant orientation is generally visible, determining a precise local orientation at specific points remains challenging. In addition, raters faced difficulties in defining structure boundaries, particularly in deciding whether to segment a small bundle with localized orientation and width or a larger region with overall orientation and width. This variation is reflected in the boxplot of Fig. 9(b). Finally, the results for the THG data representing connective tissue are presented in Fig. 9(c). The mean accuracy of the detected structures reached 88.8%, with angle and width detection accuracies of 85.7% and 92%, respectively. Variability in the rater-generated masks can be attributed to the subjective interpretation of what constitutes signal versus background. Discrepancies in angle measurements stem from the nature of the tissue; in a sinusoidal wave pattern, orientation changes sharply at each point, making correct labeling difficult. By contrast, width labels showed strong agreement likely due to the high image quality. Compared with blood vessels, the structures have sharper edges and a more consistent width, making them easier to delineate.

## 5 Discussion

Understanding the dynamic remodeling of biological tissues requires imaging techniques capable of capturing structural changes in real time. Unlike *ex vivo* methods, which provide high-resolution but static snapshots of tissue architecture, *in vivo* imaging enables the observation of biological processes as they unfold in their native environment. A representative example is the remodeling of bone tissue after injury, particularly the reorganization of collagen and vascular networks, which continuously adapt to mechanical forces, damage, and pathological conditions. By visualizing these dynamic processes in living systems, *in vivo* imaging provides essential insights into how tissues respond and reorganize under physiological and pathological stimuli.

However, extracting precise structural information from *in vivo* imaging remains challenging due to inherent technological limitations and the complexity of biological tissues. Intravital optical imaging techniques, although powerful, introduce noise and artifacts, making it difficult to obtain direct, high-fidelity measurements of structural orientation and tissue organization. Given these limitations, computational image analysis tools are essential for extracting meaningful data.

Quantifying structural orientation, particularly collagen fiber alignment, is a key focus of many research studies. Nejim et al.^18^ provided a comprehensive overview of available tools for this purpose, along with a comparative analysis of various methods applied to a representative SHG image.

A common strategy involves analyzing specific regions of interest (ROIs) using the Fourier transform (FT). The resulting spectra are processed differently depending on the study’s goals:

• Binarization and line fitting: Rao et al.^37^ binarized the FT spectra and estimated the dominant orientation by fitting a line. The standard deviation of angles across multiple ROIs is used to quantify collagen fiber alignment.
• Ellipse fitting: other studies, such as Refs. 14 and 38, fit an ellipse to the FT spectrum and use the axis ratio as a metric to quantify the degree of fiber disorder. In addition to this frequency-domain analysis, Zeitoune et al.^38^ also applied the GLCM analysis directly to the ROIs to extract texture-based spatial information.
• Grid-based global orientation mapping: to capture orientation across the entire image, Sivaguru et al.^13^ and Salazar Coariti et al.^39^ overlaid a grid and compute orientation within each cell by fitting lines to binarized FT spectra. This provides a spatially resolved, global view of collagen alignment. In addition, Salazar Coariti et al. proposed a novel metric based on fluid mechanics variables, offering a unique and interdisciplinary approach to fiber orientation analysis.

Unlike the FT-based studies, Hu et al.^22^ used a texture analysis approach that does not rely on the frequency domain. They developed an orientation-dependent GLCM, which enhances the standard method by incorporating directionality. This allows for more accurate detection of dominant fiber orientations directly from spatial pixel relationships in the image.

Orientation can also be derived from image gradients, as implemented in the Fiji plugin OrientationJ.^40^ This method identifies the direction of maximum intensity change, corresponding to dominant structural orientations.^41^

Beyond traditional frequency- and texture-based approaches, several advanced and hybrid methods have been developed to improve fiber orientation analysis. One such method is the CT-FIRE algorithm,^17^ which combines a curvelet transform (CT)-based denoising filter with the FIRE fiber extraction algorithm.^42^ The CT^43^ surpasses wavelets in detecting lines and edges, effectively enhancing fiber boundaries.

In parallel, CNNs have emerged as powerful tools for image analysis. However, their application to collagen imaging remains limited^44^^,^^45^ largely due to the challenges of acquiring accurately labeled datasets. Manual annotation is time-consuming, susceptible to inter-rater variability—as observed in our own annotations—and impractical for large datasets. These limitations underscore the need for robust, data-efficient learning approaches that can perform well with minimal supervision.

Although existing methods provide valuable insights, the complexity of *in vivo* data required a different approach. To address these challenges, we developed a Radon transform–based algorithm with embedded image-patching, which demonstrated high accuracy and adaptability across different imaging modalities. It achieved mean structure detection accuracies of 96.2% for 2PM, 89.7% for SHG, and 88.8% for THG data. Angle and width detection accuracies varied across datasets, with the highest consistency observed in THG images (85.7% and 92%, respectively) and greater discrepancies in SHG data, likely due to the complexity of collagen bundle structures.

These results emphasize the advantage of a mathematically driven algorithm that enables objective image analysis, minimizing reliance on manual labeling, which is often time-consuming, inconsistent, and prone to subjective biases. The algorithm performed particularly well on 2PM images, achieving high accuracy in structure and angle detection compared with individual raters. By contrast, SHG images posed greater challenges due to the subjective nature of collagen bundle delineation, leading to higher discrepancies in orientation labeling. Nevertheless, the algorithm maintained competitive accuracy despite these complexities. Results on THG data further demonstrated its ability to detect structures with well-defined boundaries and accurately estimate widths.

A key strength of the algorithm is its robustness across diverse imaging conditions, effectively handling noise and structural variations while maintaining high accuracy. In contrast to Fourier transform–based approaches, which can yield varying results depending on factors such as threshold values for binarizing the spectrum, the thresholding function, morphological operations, and fitting functions, our method simplifies the process by requiring only the selection of the background value—easily extractable from the image. GLCM-based methods, although powerful for analyzing textural features, are sensitive to noise, which can lead to variability in results. In comparison, our algorithm is more robust against noise, ensuring greater consistency and accuracy in orientation detection without the need for intricate parameter adjustments. In addition, the patch-based analysis of the Radon transform (RT) enables comprehensive image examination, detecting structures at varying scales based on the specified patch dimensions. This adaptability enhances fiber detection and provides a more detailed and nuanced orientation analysis. These strengths make the algorithm a versatile and efficient tool for orientation analysis. Minimal preprocessing and parameter tuning simplify the workflow, ensuring that results are not overly dependent on initial processing steps.

Despite these strengths, the algorithm has certain limitations. Although the patch-based analysis enables a comprehensive examination of the entire image, it is also the primary source of errors. The structures detected depend on the patch size: finer patch sizes allow the detection of finer structures. However, if the patch size becomes too small relative to the underlying structure it slides over, the orientation information is lost. In addition, errors occurring at the edges are associated with the patch’s position. If the patch “cuts” the underlying structure at its edge, the algorithm will detect the orientation corresponding to the maximum number of pixels aligned in that direction within the patch, potentially leading to inaccuracies. Another challenge lies in selecting the threshold value for the background, which is not always straightforward. For example, in the SHG data of the LIMBostomy, determining the optimal background threshold proved difficult. Future improvements will address these challenges by integrating the algorithm’s output with a secondary analysis method—such as a statistical approach or an alternative transformation—to filter out errors and validate correct results.

Despite these limitations, the algorithm strikes a valuable balance between simplicity, robustness, and effectiveness, offering a reliable solution for orientation analysis across diverse imaging contexts. We expect this to be particularly useful for the *in vivo* imaging community, especially given the growing interest in studying living biomechanics and tissue architecture in a dynamic, high-resolution manner.

## 6 Conclusion and Outlook

The algorithm holds significant promise for tissue structural arrangement analysis, particularly in its ability to process images acquired with various modalities without introducing artifacts from specific preprocessing or segmentation techniques. For instance, in LIMBostomy^31^ data, the algorithm can quantify the orientation and arrangement of blood vessels and collagen bundles following a bone fracture. This capability can be pivotal in assessing the stages of tissue regeneration and understanding how different tissues influence one another during the healing process. To further enhance its functionality, future improvements could integrate a skeletonization function that connects all points belonging to a single fiber. This addition would allow for the assessment of both the width and orientation of fibers along their entire length, providing deeper insights into tissue structure and improving the understanding of complex biological processes. In addition, an important future development could involve extending the algorithm to analyze 3D data, enabling a deeper understanding of tissue networks and their organization in three-dimensional space. This would be particularly valuable for capturing the full complexity of biological tissues and their structural relationships. In summary, the proposed algorithm provides a robust, flexible, and efficient solution for structural orientation analysis in biological images. By addressing challenges such as noise sensitivity and reducing the need for extensive preprocessing, it advances imaging analysis while maintaining simplicity and adaptability across multiple modalities. Its patch-based approach facilitates comprehensive analysis of the entire image, offering a global perspective on structural orientation.

## Supplementary Material

10.1117/1.JBO.30.8.086001.s01

## Data Availability

The archived version of the code described in this paper and the data presented are freely available at the following GitHub repository: RadonTransform.

## References

[1] Lackington W. A. Thompson K., “Fracture healing and progress towards successful repair,” in Racing for the Surface: Antimicrobial and Interface Tissue Engineering, Li B., Eds., pp. 225–243, Springer, Cham (2020).

[2] Filipowska J. et al., “The role of vasculature in bone development, regeneration and proper systemic functioning,” Angiogenesis 20, 291–302 (2017). 10.1007/s10456-017-9541-1 28194536 PMC5511612

[3] Wang X. et al., “The role of collagen in determining bone mechanical properties,” J. Orthop. Res. 19(6), 1021–1026 (2001). JOREDR 0736-0266 10.1016/S0736-0266(01)00047-X 11781000

[4] Sun B., “The mechanics of fibrillar collagen extracellular matrix,” Cell Rep. Phys. Sci. 2(8), 100515 (2021). 10.1016/j.xcrp.2021.100515 34485951 PMC8415638

[5] Golaraei A. et al., “Changes of collagen ultrastructure in breast cancer tissue determined by second-harmonic generation double stokes-Mueller polarimetric microscopy,” Biomed. Opt. Express 7(10), 4054–4068 (2016). BOEICL 2156-7085 10.1364/BOE.7.004054 27867715 PMC5102540

[6] Sadeghinia M. J. et al., “Quantified planar collagen distribution in healthy and degenerative mitral valve: biomechanical and clinical implications,” Sci. Rep. 14(1), 15670 (2024). SRCEC3 2045-2322 10.1038/s41598-024-65598-w 38977735 PMC11231298

[7] Pantazis P. et al., “Second harmonic generating (SHG) nanoprobes for in vivo imaging,” Proc. Natl. Acad. Sci. 107(33), 14535–14540 (2010). 10.1073/pnas.1004748107 20668245 PMC2930484

[r8] Zoumi A. Yeh A. Tromberg B. J., “Imaging cells and extracellular matrix in vivo by using second-harmonic generation and two-photon excited fluorescence,” Proc. Natl. Acad. Sci. 99(17), 11014–11019 (2002). 10.1073/pnas.172368799 12177437 PMC123202

[9] Niesner R. A. Hauser A. E., “Recent advances in dynamic intravital multi-photon microscopy,” Cytom. Part A 79(10), 789–798 (2011).1552-4922 10.1002/cyto.a.21140 21905212

[10] Holota R. Němeček S., “Recognition of oriented structures by 2D Fourier transform,” Appl. Electron., 88–92 (2002). DJYID7 0258-7998

[11] Pijanka J. K. et al., “Quantification of collagen fiber structure using second harmonic generation imaging and two-dimensional discrete Fourier transform analysis: application to the human optic nerve head,” J. Biophotonics 12(5), e201800376 (2019). 10.1002/jbio.201800376 30578592 PMC6506269

[12] Rao R. A. et al., “Quantitative analysis of forward and backward second-harmonic images of collagen fibers using Fourier transform second-harmonic-generation microscopy,” Opt. Lett. 34(24), 3779–3781 (2009). OPLEDP 0146-9592 10.1364/OL.34.003779 20016611

[13] Sivaguru M. et al., “Quantitative analysis of collagen fiber organization in injured tendons using Fourier transform-second harmonic generation imaging,” Opt. Express 18(24), 24983–24993 (2010). OPEXFF 1094-4087 10.1364/OE.18.024983 21164843

[14] Wang Y. et al., “Diagnosing temporomandibular joint disorders using second harmonic imaging of collagen fibers,” J. Biophotonics 15(10), e202200075 (2022). 10.1002/jbio.202200075 35588374

[15] Farge M. et al., “Wavelet transforms and their applications to turbulence,” Annu. Rev. Fluid Mech. 24(1), 395–458 (1992). ARVFA3 0066-4189 10.1146/annurev.fl.24.010192.002143

[16] Candes E. J. Donoho D. L., “Continuous curvelet transform: I. Resolution of the wavefront set,” Appl. Comput. Harmonic Anal. 19(2), 162–197 (2005). ACOHE9 1063-5203 10.1016/j.acha.2005.02.003

[17] Bredfeldt J. S. et al., “Computational segmentation of collagen fibers from second-harmonic generation images of breast cancer,” J. Biomed. Opt. 19(1), 016007 (2014). JBOPFO 1083-3668 10.1117/1.JBO.19.1.016007 24407500 PMC3886580

[18] Nejim Z. et al., “Quantitative analysis of second harmonic generated images of collagen fibers: a review,” Res. Biomed. Eng. 39(1), 273–295 (2023). 10.1007/s42600-022-00250-y

[19] Adams R. Bischof L., “Seeded region growing,” IEEE Trans. Pattern Anal. Mach. Intell. 16(6), 641–647 (1994). ITPIDJ 0162-8828 10.1109/34.295913

[20] Otsu N. et al., “A threshold selection method from gray-level histograms,” Automatica 11(285–296), 23–27 (1975). ATCAA9 0005-1098 10.1109/TSMC.1979.4310076

[21] Berg S. et al., “ilastik: interactive machine learning for (bio)image analysis,” Nat. Methods 16, 1226–1232 (2019).1548-7091 10.1038/s41592-019-0582-9 31570887

[22] Hu W. et al., “Characterization of collagen fibers by means of texture analysis of second harmonic generation images using orientation-dependent gray level co-occurrence matrix method,” J. Biomed. Opt. 17(2), 026007 (2012). JBOPFO 1083-3668 10.1117/1.JBO.17.2.026007 22463039

[23] Cicchi R. et al., “Extraction of collagen morphological features from second-harmonic generation microscopy images via GLCM and CT analyses: a cross-laboratory study,” J. Biophotonics 17(8), e202400090 (2024). 10.1002/jbio.202400090 38937995

[24] Radon J., “On the determination of functions from their integrals along certain manifolds,” Mathematisch-Physische Klasse 69, 262–277 (1917).

[25] Deans S. R., The Radon Transform and Some of its Applications, Courier Corporation (2007).

[r26] Elouedi I. et al., “Generalized multi-directional discrete Radon transform,” Signal Process. 93(1), 345–355 (2013). 10.1016/j.sigpro.2012.07.031

[r27] Toft P. A., “The Radon transform-theory and implementation,” PhD Thesis, Technical Univ. of Denmark (1996).

[r28] Milanfar P., “A model of the effect of image motion in the Radon transform domain,” IEEE Trans. Image Process. 8(9), 1276–1281 (1999). IIPRE4 1057-7149 10.1109/83.784439 18267544

[r29] Baby D. Devaraj S. J. Raj M. A., “Leukocyte classification based on statistical measures of Radon transform for monitoring health condition,” Biomed. Phys. Eng. Express 7(6), 065031 (2021). 10.1088/2057-1976/ac2e16 34624876

[30] McLean J. P. et al., “High-speed collagen fiber modeling and orientation quantification for optical coherence tomography imaging,” Opt. Express 27(10), 14457–14471 (2019). OPEXFF 1094-4087 10.1364/OE.27.014457 31163895 PMC6825605

[31] Stefanowski J. et al., “Limbostomy: longitudinal intravital microendoscopy in murine osteotomies,” Cytom. Part A 97(5), 483–495 (2020).1552-4922 10.1002/cyto.a.23997 32196971

[32] Rakhymzhan A. et al., “Coregistered spectral optical coherence tomography and two-photon microscopy for multimodal near-instantaneous deep-tissue imaging,” Cytom. Part A 97(5), 515–527 (2020).1552-4922 10.1002/cyto.a.24012 32293804

[33] Rakhymzhan A. et al., “Optimized intravital three-photon imaging of intact mouse tibia links plasma cell motility to functional states,” Iscience 27(10), 110985 (2024). 10.1016/j.isci.2024.110985 39391739 PMC11466647

[34] Bias A. et al., “Multimodal multiphoton dynamic imaging: determining the force field in the mouse paw,” Proc. SPIE 12834, 1283403 (2024). PSISDG 0277-786X 10.1117/12.3001601

[35] Reismann D. et al., “Longitudinal intravital imaging of the femoral bone marrow reveals plasticity within marrow vasculature,” Nat. Commun. 8(1), 2153 (2017). NCAOBW 2041-1723 10.1038/s41467-017-01538-9 29255233 PMC5735140

[36] Ramesh G. Srinivasa N. Rajgopal K., “An algorithm for computing the discrete Radon transform with some applications,” in Fourth IEEE Region 10 Int. Conf. TENCON, IEEE, pp. 78–81 (1989). 10.1109/TENCON.1989.176899

[37] Rao R. A. R. Mehta M. R. Toussaint K. C. Jr., “Fourier transform-second-harmonic generation imaging of biological tissues,” Opt. Express 17(17), 14534–14542 (2009). OPEXFF 1094-4087 10.1364/OE.17.014534 19687932

[38] Zeitoune A. A. et al., “Epithelial ovarian cancer diagnosis of second-harmonic generation images: a semiautomatic collagen fibers quantification protocol,” Cancer Inform. 16, 117693511769016 (2017). 10.1177/1176935117690162 PMC5392028 28469386

[39] Salazar Coariti A. C. et al., “Fluid mechanics approach to analyzing collagen fiber organization,” J. Biomed. Opt. 27(1), 016503 (2022). JBOPFO 1083-3668 10.1117/1.JBO.27.1.016503 35102730 PMC8802803

[40] Schindelin J. et al., “Fiji: an open-source platform for biological-image analysis,” Nat. Methods 9(7), 676–682 (2012).1548-7091 10.1038/nmeth.2019 22743772 PMC3855844

[41] Püspöki Z. et al., “Transforms and operators for directional bioimage analysis: a survey,” in Focus on Bio-image Informatics, De Vos W. Munck S. Timmermans J. P., Eds., pp. 69–93, Springer, Cham (2016). 10.1007/978-3-319-28549-8_3 27207363

[42] Stein A. M. et al., “An algorithm for extracting the network geometry of three-dimensional collagen gels,” J. Microsc. 232(3), 463–475 (2008). JMICAR 0022-2720 10.1111/j.1365-2818.2008.02141.x 19094023

[43] Candes E. J. Guo F., “New multiscale transforms, minimum total variation synthesis: applications to edge-preserving image reconstruction,” Signal Process. 82(11), 1519–1543 (2002). 10.1016/S0165-1684(02)00300-6

[44] Schmarje L. et al., “2D and 3D segmentation of uncertain local collagen fiber orientations in SHG microscopy,” Lect. Notes Comput. Sci. 11824, 374–386 (2019). LNCSD9 0302-9743 10.1007/978-3-030-33676-9_26

[45] Liang L. Liu M. Sun W., “A deep learning approach to estimate chemically-treated collagenous tissue nonlinear anisotropic stress-strain responses from microscopy images,” Acta biomaterialia 63, 227–235 (2017). 10.1016/j.actbio.2017.09.025 28939354 PMC5653437

