# Supplementary material for: Segmentation-free Radon transform algorithm to detect orientation and size of tissue structures in multiphoton microscopy images
Source: J Biomed Opt. 2025 Aug 4;30(8):086001. doi: 10.1117/1.JBO.30.8.086001 (PMC12322599; doi:10.1117/1.JBO.30.8.086001)
Supplement: Supplementary file 1 [file JBO_030_086001_SD001.pdf]

Supplementary information

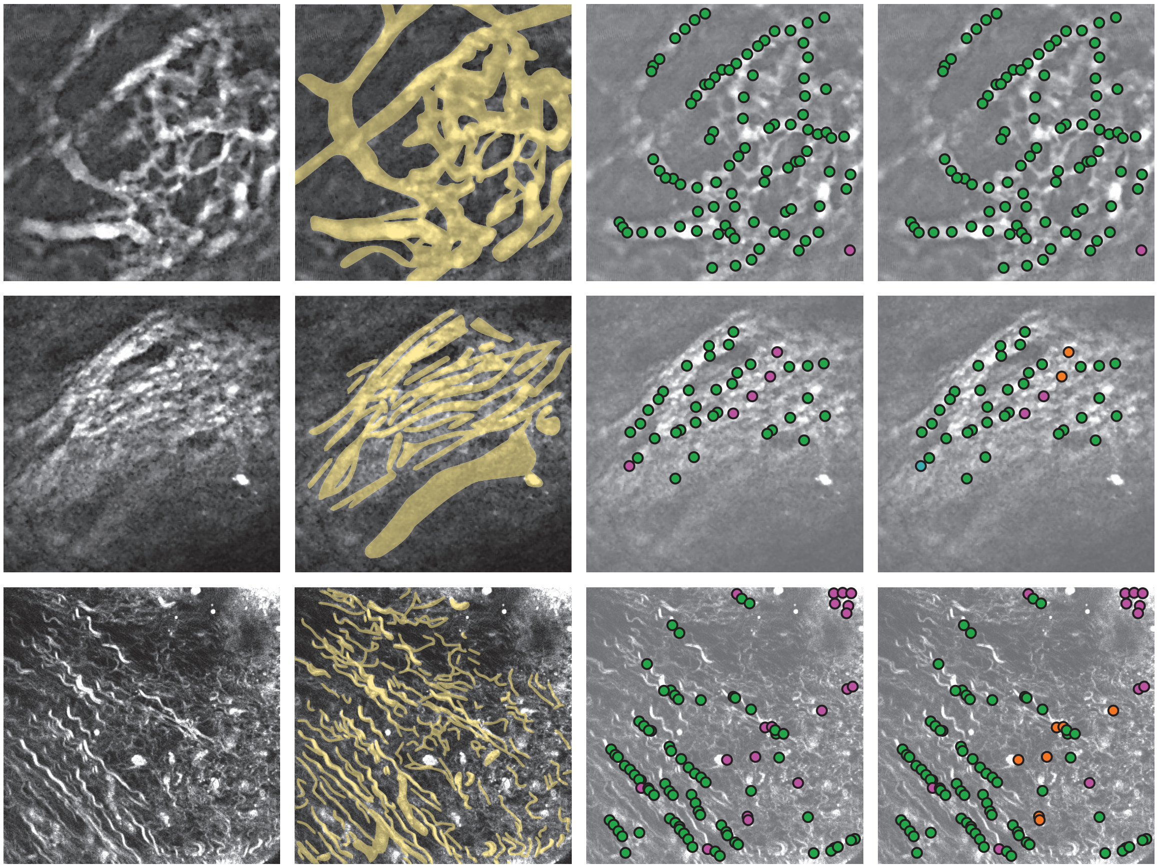

(a) (b) (c) (d)

Fig S1 Metrics for evaluating the position of detected points. (a) Input images, showing blood vessels, collagen bundles, and connective tissue from top to bottom. (b) Each labeler manually drew a mask for the input image. (c) A detected point was classified as correct if it fell within the mask. Correct points are marked in green, while points outside the mask are marked in magenta. (d) The raters reviewed the magenta points and determined if any should be reclassified as correct. These newly added points are marked in orange.

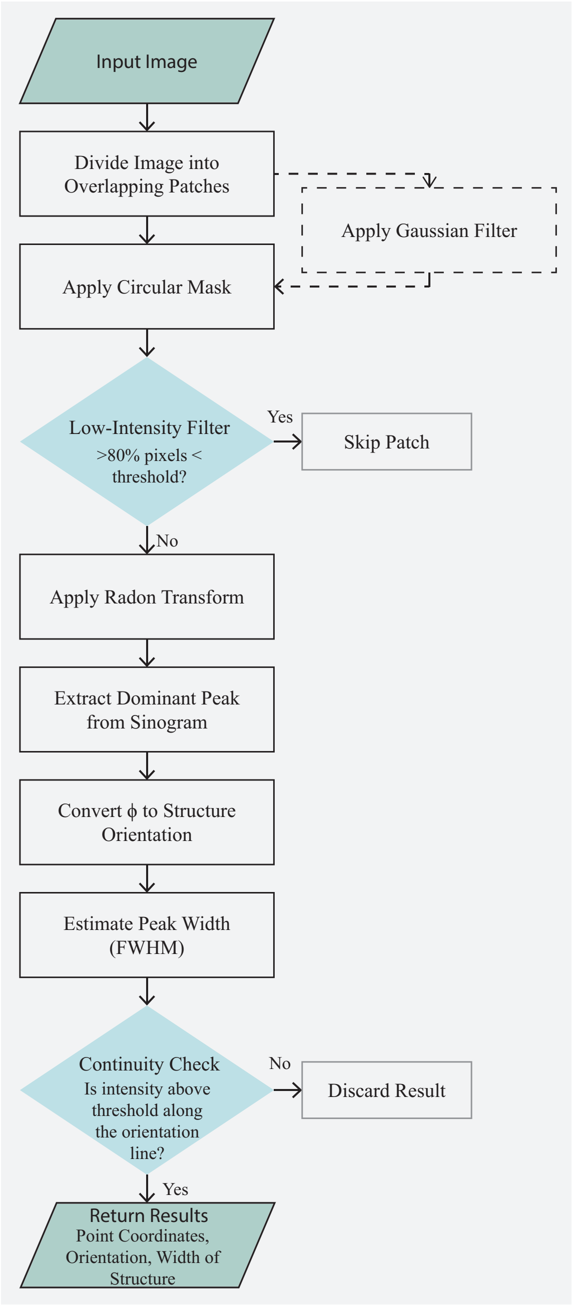

Fig S2 Flowchart of the proposed Radon Transform based algorithm for detecting oriented structures in images. The diagram outlines the sequential steps, including patch generation, background filtering, Radon transform application, peak detection, and final filtering based on intensity profile analysis.

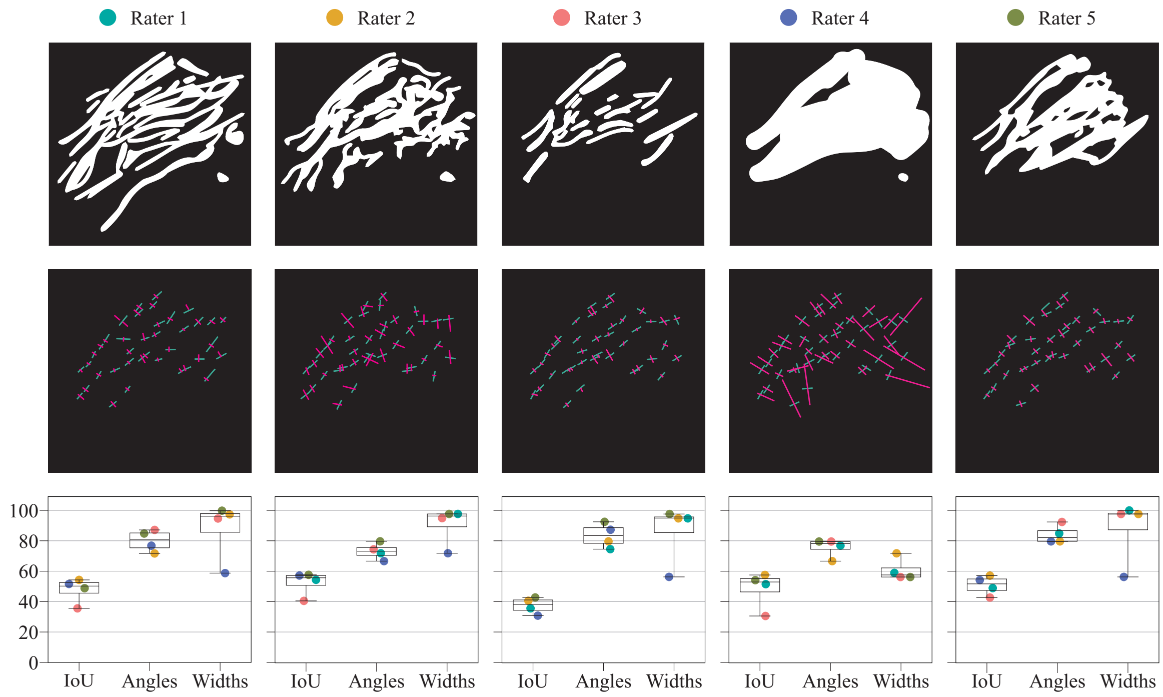

Fig S3 Variability in rater annotations for the SHG data. The first row displays the masks drawn by different raters, while the second row shows the orientation (green lines) and width (magenta) assigned by each rater. Differences in annotations are evident due to the complexity of the image, the structural intricacies, and the challenge of defining the boundaries of a collagen bundle. In each column, the corresponding rater is treated as the ground truth. The last row presents the evaluation results of the other raters relative to the reference rater, using the Intersection over Union (IoU) metric for the masks and the differences between the orientations and widths assigned by the reference rater and the other raters.

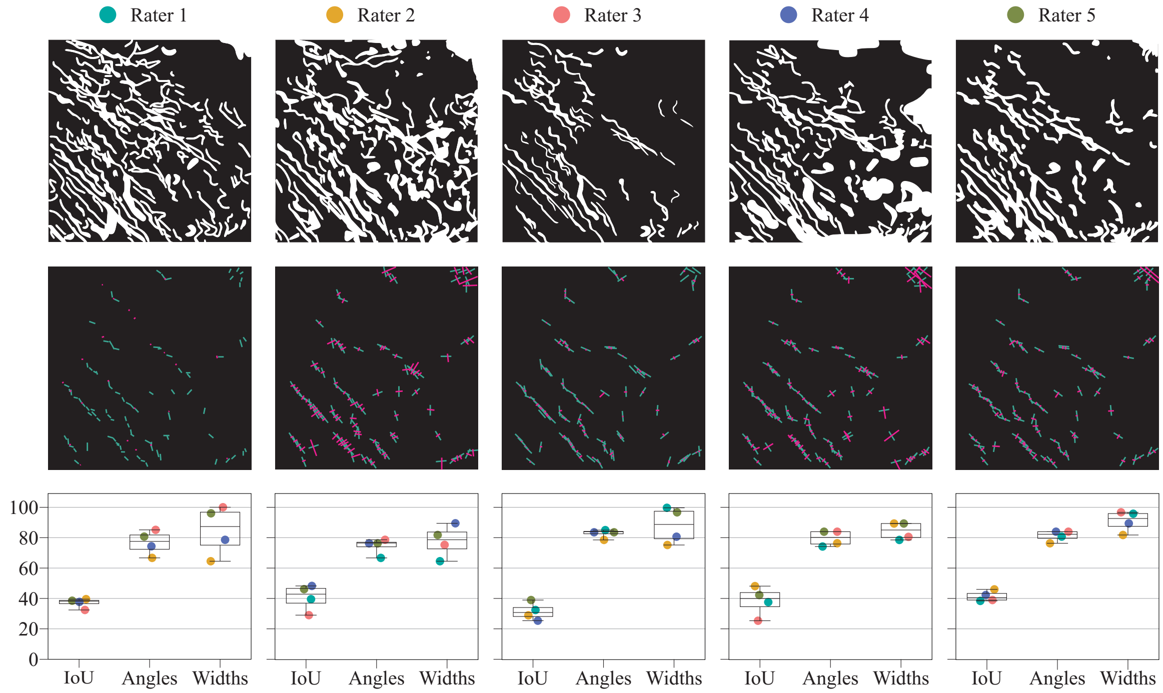

Fig S4 Variability in rater annotations for the THG data. The first row displays the masks drawn by different raters, while the second row shows the orientation (green lines) and width (magenta) assigned by each rater. Differences in annotations are evident due to the complexity of the image, the challenge of determining the correct angle for a rapidly changing orientation, such as a wavy structure, and the subjectivity involved in distinguishing signal from background. In each column, the corresponding rater is treated as the ground truth. The last row presents the evaluation results of the other raters relative to the reference rater, using the Intersection over Union (IoU) metric for the masks and the differences between the orientations and widths assigned by the reference rater and the other raters.

Table S1 Refined Point Detection Accuracy. Final adjusted accuracy of detected points following the re-evaluation process. Initially detected correct points are listed alongside additional points verified as correct after reassessment. While only a small number of additional points were included, this refinement ensured the most precise accuracy assessment with minimal impact on overall results.

Data	Rater	First Check			Second Check		
		Points	Angles	Width	Points	Angles	Width
2PM	01	98.88	85.23	79.55	98.88	85.23	79.55
	02	94.38	84.52	88.10	94.38	84.52	88.10
	03	94.38	83.33	84.52	96.63	83.72	82.56
	04	95.51	78.82	88.24	95.51	78.82	88.24
	05	95.51	87.06	92.94	95.51	87.06	92.94
SHG	01	89.74	82.86	88.57	94.87	81.08	86.49
	02	92.31	69.44	88.89	92.31	69.44	88.89
	03	53.85	100.00	85.71	71.79	92.86	85.71
	04	97.44	63.16	71.05	97.44	63.16	71.05
	05	84.62	90.91	90.91	92.31	88.89	91.67
THG	01	83.87	78.21	91.03	90.32	78.57	91.67
	02	96.77	82.22	88.89	96.77	82.22	88.89
	03	70.97	96.97	96.97	70.97	96.97	96.97
	04	90.32	85.71	90.48	90.32	85.71	90.48
	05	93.55	85.06	91.95	95.70	85.39	92.13
